# Golden orb-weaving spider (*Trichonephila clavipes*) silk genes with sex-biased expression and atypical architectures

**DOI:** 10.1093/g3journal/jkaa039

**Published:** 2020-12-22

**Authors:** Sandra M Correa-Garhwal, Paul L Babb, Benjamin F Voight, Cheryl Y Hayashi

**Affiliations:** 1 Division of Invertebrate Zoology and Sackler Institute for Comparative Genomics, American Museum of Natural History, New York, NY 10024, USA; 2 Department of Systems Pharmacology and Translational Therapeutics, Perelman School of Medicine, University of Pennsylvania, Philadelphia, PA 19104, USA; 3 Department of Genetics, Perelman School of Medicine at the University of Pennsylvania, Philadelphia, PA 19104, USA; 4 Institute for Translational Medicine and Therapeutics, Perelman School of Medicine at the University of Pennsylvania, Philadelphia, PA 19104, USA

**Keywords:** spidroins, differential gene expression, spider silk

## Abstract

Spider silks are renowned for their high-performance mechanical properties. Contributing to these properties are proteins encoded by the spidroin (spider fibroin) gene family. Spidroins have been discovered mostly through cDNA studies of females based on the presence of conserved terminal regions and a repetitive central region. Recently, genome sequencing of the golden orb-web weaver, *Trichonephila clavipes*, provided a complete picture of spidroin diversity. Here, we refine the annotation of *T. clavipes* spidroin genes including the reclassification of some as non-spidroins. We rename these non-spidroins as spidroin-like (SpL) genes because they have repetitive sequences and amino acid compositions like spidroins, but entirely lack the archetypal terminal domains of spidroins. Insight into the function of these spidroin and SpL genes was then examined through tissue- and sex-specific gene expression studies. Using qPCR, we show that some silk genes are upregulated in male silk glands compared to females, despite males producing less silk in general. We also find that an enigmatic spidroin that lacks a spidroin C-terminal domain is highly expressed in silk glands, suggesting that spidroins could assemble into fibers without a canonical terminal region. Further, we show that two SpL genes are expressed in silk glands, with one gene highly evolutionarily conserved across species, providing evidence that particular SpL genes are important to silk production. Together, these findings challenge long-standing paradigms regarding the evolutionary and functional significance of the proteins and conserved motifs essential for producing spider silks.

## Introduction

Spider webs are among the most striking animal architectures on the planet. Much attention has focused on the outstanding mechanical properties of spider silks, with the ultimate goal of understanding the structure: function relationships that underlie these properties. Through genomic studies, we are realizing that the roles, biological functions, and even what historically we have thought of as relevant to silk production are changing. For example, the large orb-webs that have been widely studied are built by female spiders. In contrast, how males utilize silk is less well understood. Recently, the genome and multiple tissue transcriptomes of the golden orb-weaver were published, in which full-length silk genes were characterized along with tissue-specific gene expression profiles (Babb *et al.* 2017). We now have the opportunity to better understand how different silk proteins are utilized and their potential biological functions by characterizing gene expression between sexes. 

The golden orb-web weaving spider *Trichonephila clavipes* (Linnaeus, 1767) [formerly *Nephila clavipes* (Kuntner *et al.* 2019)] is a strongly sexual size dimorphic species from which the first silk complementary DNA (cDNA) was sequenced (Xu and Lewis 1990). The differences in body size have been attributed to evolutionary female gigantism or male dwarfism (Vollrath and Parker 1992; Coddington *et al.* 1997; Vollrath 1998; Danielson-François *et al.* 2012). Male and female spiders also show differences in behaviors and silk use. For example, after sexual maturation, male spiders cease web construction for prey capture and instead adopt a roving lifestyle in search of receptive females, while female spiders spend more time and energy in egg-case production. These sex-specific behavioral differences affect the silk types a spider uses. For instance, as males move from place to place, they produce draglines or safety lines (made of major and minor ampullate silk) and attachment silk (pyriform silk) to travel on and secure themselves (Escalante and Masís-Calvo 2014; Correa-Garhwal *et al.* 2017). Moreover, *T. clavipes* males lack the morphological apparatus to produce silks related to web construction such as aggregate and flagelliform silk spigots (Moore 1977; Murphy and Roberts 2015). One way to quantify silk use is by measuring gene transcript levels; however, relatively few studies have measured and compared male and female silk gene expression (Correa-Garhwal *et al.* 2017, 2018, 2019). Notably, Babb *et al.* (2017) presented gene expression profiles for *T. clavipes* females but not males.

Orb-web weaving spiders such as *T. clavipes* produce seven different types of silk that are used for diverse ecological purposes. Each of the different silks is associated with its own specialized type of silk gland: prey-wrapping fibers emerge from aciniform glands, attachment silk from pyriform glands, safety draglines from major ampullate glands, temporary capture spiral silk from minor ampullate glands, capture spiral filament from flagelliform glands, sticky glue from aggregate glands, and egg case coverings from tubuliform glands. Within each silk gland is a pool of silk proteins, of which the dominant proteins are spidroins (a contraction of “spider fibroins”; Hinman and Lewis 1992).

Spidroins are a family of large proteins with non-repetitive amino (N) and carboxyl (C) terminal domains that flank a large repetitive region of amino acid sequences (Gatesy *et al.* 2001; Ayoub *et al.* 2007; Garb *et al.* 2010; Chaw *et al.* 2016; Clarke *et al.* 2017). In addition to spidroins, Babb *et al.* (2017) expanded the silk gene set in *T. clavipes* to include genes that have affinities to the repetitive regions of spidroins, but elude assignment to known spidroin types. Here, we refine the annotation of these sequences to “spidroin-like” (SpL) since they lack the expected spidroin terminal regions. The absence of spidroin terminal regions runs counter to the established theory that the conserved terminal regions are required for silk processing (Beckwitt and Arcidiacono 1994; Huemmerich *et al.* 2004; Sponner *et al.* 2004; Ittah *et al.* 2007; Gaines *et al.* 2010; Gao *et al.* 2013; Otikovs *et al.* 2015).

In this study, we investigate the tissue- and sex-specific expression of spidroin and SpL genes in both male and female *T. clavipes*. We find that most silk genes have sex-specific expression patterns and that some are expressed in unexpected locations, such as SpL genes with significantly higher expression in male pedipalps. We also assess the extent to which each spidroin and SpL sequence is conserved across species or unique to the *T. clavipes* lineage. We find the SpL gene (*SpL_1339*) to be highly conserved across multiple spider species, indicating the functional and evolutionary significance of an SpL in silk production. By contrast, we show that the spidroin C-terminal domain, which has conserved motifs thought to be important fiber formation (Beckwitt and Arcidiacono 1994; Huemmerich *et al.* 2004; Sponner *et al.* 2004; Ittah *et al.* 2007; Gaines *et al.* 2010; Gao *et al.* 2013; Otikovs *et al.* 2015), can be entirely lost, as seen in the spidroin Sp_5803.

## Materials and methods

### Samples and specimen preparation

*Trichonephila clavipes* (Linnaeus, 1767) was used for two sets of experiments: male expression analysis and the male-female comparative expression analysis, each using different collections of samples. For the male expression analysis, RNA was extracted from four wild-caught *T. clavipes* adult males (Nep021–024) collected from Charleston County, South Carolina, USA (Supplementary Table S1). For the male–female comparative expression analysis, RNA was extracted from three wild-caught adult males (Nep028–030) and three wild-caught adult females (Nep025–027), also collected from Charleston County, South Carolina, USA.

For the male-specific expression analysis, microdissections were performed on the four adult males (Nep021–024) used for the downstream male-specific expression analysis. Each biological replicate was anesthetized with CO_2_, then the abdomen was separated from the cephalothorax. From the abdomen, nonsilk gland and spinneret tissue was carefully removed. Silk glands that could be identified by relative position and morphology were individually collected by severing their ducts near the spinnerets. Pedipalps and legs were removed and combined into a single tissue isolate. Venom glands were collected after separation of the chelicerae from the cephalothorax, and the remaining cephalothorax tissue was retained as the “cephalothorax” sample. In this manner, samples Nep021 and Nep023 had individual tissue-specific subsamples of pedipalps and legs, venom glands, cephalothorax (no venom), major ampullate silk glands, minor ampullate silk glands, and “small silk glands” (aciniform and pyriform silk glands). For male samples Nep022 and Nep024, the tissue-specific subsamples for pedipalps and legs, venom glands, cephalothorax (no venom), and “total silk glands” (collection of major ampullate, minor ampullate, aciniform, and pyriform silk glands) were collected (Supplementary Table S1; Supplementary File S1, tab: “Samples”). In total, 20 unique tissues were obtained from four males for the male-specific expression analysis.

To create pairings of comparable tissue types for the male–female comparative expression analysis, dissections were performed on the remaining three adult male individuals (Nep028–030) and three adult female individuals (Nep025–027). For each male sample, the following four tissues were isolated using sterile techniques, and processed individually: pedipalps, legs, cephalothorax, and abdomen. Due to the much larger sizes of female *T. clavipes* females compared to males, female tissues required further division for efficient RNA extraction and purification: pedipalps, legs, chelicerae, anterior cephalothorax (no pedipalps or chelicerae), posterior cephalothorax, anterior abdomen, and posterior abdomen. Importantly, once extracted and purified (see below), the RNA extracts from the female tissues were then recombined for each individual on the basis of anatomy to better approximate male tissue subsections: pedipalps, legs, “cephalothorax” (combination of RNA from chelicerae, anterior cephalothorax, and posterior cephalothorax subsections), and “abdomen” (combination of RNA from anterior and posterior abdomen subsections). In this way, each individual from either sex thus had a total of four RNA extracts that would subsequently be processed into cDNA via reverse transcription, and then assayed by quantitative PCR (qPCR) to assess relative gene expression in the male–female comparative analysis (Supplementary File S2; Supplementary File S1, tab: “Samples”).

### RNA extraction

All samples were transferred into individual 2 ml Eppendorf Safe-Lock microcentrifuge tubes containing 1 ml RNAlater (Life Technologies) and spun at 5000 × *g* for 5 min to pellet the tissues. RNA was extracted using the following TRIzol extraction method. RNAlater supernatants (and residual salt crystals) were removed from each sample and archived. Each tube then received 500 μl TRIzol (Life Technologies) and one sterile 5 mm stainless steel ball bearing (Qiagen). Tubes were racked into frozen TissueLyser Adapter blocks (Qiagen) and loaded onto a TissueLyser II (Qiagen) for automated sample disruption and homogenization. High-speed shaking was carried out at 30 Hz for three minutes at room temperature. Each sample was then transferred to 2 ml “light” phase lock gel tubes (5Prime).

Next, 100 μl of chloroform (Macron) was added to each sample, and samples spun at 10,000 RPM for 10 min at 4°C in a pre-chilled microcentrifuge (Eppendorf). The RNA-containing aqueous phase of each sample was transferred to a new clean tube, combined with two volumes of 100% ethanol, and mixed gently. Samples were purified using the RNeasy Mini Kit spin columns (Qiagen). All samples then underwent a secondary cleanup step using the RNA Clean & Concentrator-5 kit, and which included DNAse I treatment (Zymo Research). Small aliquots (∼5 μl) were quickly pulled from each of the 44 RNA extractions (20 for the male-specific analysis, 24 for the male-female comparative analysis) for quality control and quantification experiments, and the remaining stock extracts were immediately stored at −80°C. Finally, DNA removal was confirmed via 1.2% TAE agarose gel.

### Quantitative PCR analysis

To test relative expression of loci in discrete anatomical subsections qPCR analysis was performed. cDNA was produced from each RNA sample (0.5 μg RNA input per sample) with a High Capacity cDNA Reverse Transcription kit (Life Technologies) and run alongside multiple “noRNA/no template” [NT] and “no reverse transcriptase” [NRT] negative controls. Primers were designed to target 30 loci [ 24 spidroins, four spidroin-like, one venom locus (CRiSP/Allergen/*PR-1*), one housekeeping gene (*RPL13a* (Scharlaken *et al.* 2008))] as well as for 22 genomic-scaffold-controls for all single-exon spidroin genes (spidroins as per Babb *et al*. 2017; Supplementary File S1). After running dilutions series of the *RPL13a* housekeeping gene (1:1 to 1:10,000), qPCR reactions were set up in triplicate at 1:100 concentrations of cDNA for each tissue replicate and control versus each of the 52 targets (6864 reactions total), and their abundance was measured using SYBR Green PCR Master Mix (Life Technologies) on a ViiA 7 Real-Time PCR machine using a 5 μl protocol with 40 annealing cycles at 60°C. Relative transcript abundance was estimated across tissues using the 2^−ΔΔCT^ method (Livak and Schmittgen 2001).

### Statistical methods

To assess the relative expression levels of loci in different tissues, we calculated 2^−ΔΔCT^ values from qPCR experiments as described by Livak and Schmittgen (2001). Each gene X tissue reaction was run in triplicate (*i.e.*, three independent experiments) to control for technical variation. Cycling threshold (CT) values were averaged across technical replicates for each gene X tissue combination for each sample. The average CT values were then normalized to average *RPL13a* (housekeeping gene) CT values for the same tissue sample (ΔCT). For the male-specific expression analysis, ΔCT values for each gene X tissue combination were normalized to the ΔCT values of the same gene for the “cephalothorax” (or “head”) tissue subsection of the same sample (ΔΔCT), then raised to the negative exponent of 2 (2^−ΔΔCT^). Meanwhile, for the male–female comparative expression analysis, ΔCT values for each gene X tissue combination were normalized to the ΔCT values of the same gene for the “legs” tissue subsection of the same sample (ΔΔCT), then raised to the negative exponent of 2 (2^−ΔΔCT^). Normalization using different tissues was done to ensure legs and pedipalps were assessed as different tissues for this analysis. For all experiments, biological replicates of each tissue (from three independent spiders) were kept separate for all calculations. The variances of relative expression values for each gene were compared across tissues using F-tests, and their population means tested using one-tailed unequal-variance Wilcoxon rank sum tests. Since the hypothesis we were testing was one-directional, a one-tailed test was deemed appropriate. All F-test and Wilcoxon rank sum test input values and results are provided in Supplementary File S1, tabs: “F-Tests_MALES,” “F-Tests_BOTH_SEXES,” “Wilcoxon_MALES,” and “Wilcoxon_BOTH_SEXES.” All statistical analyses were conducted with R v3.3 (R Foundation for Statistical Computing, https://www.r-project.org/foundation/).

### Annotation of silk genes

*Trichonephila clavipes* silk sequences described by Babb *et al.* (2017) were obtained from the Whole-Genome Shotgun (WGS) database under accession MWRG00000000 (Supplementary File S3). Each gene scaffold was translated and compared to previously published *T. clavipes* spidroin sequences in Geneious (Kearse *et al.* 2012). Each silk sequence was visually examined for known spidroins characteristics such as the presence of coding regions for the conserved terminal domain regions. Further characterization was done by visually inspecting the repetitive region of each silk gene sequence and comparisons were done to assign each silk gene to a spidroin category (see *Phylogenetic analyses* section below). *Trichonephila clavipes* sequences that entirely lacked spidroin terminal domains were named SpL sequences. Each sequence was searched against the sequences described by Collin *et al.* (2018) and the non-redundant BLAST database (nr).

### Phylogenetic analyses

Amino (N)- and carboxyl (C)-terminal region encoding silk gene regions from *T. clavipes* were translated and combined with published spidroin sequences from other orb- and cob-web building species. N- and C-terminal regions were aligned separately and then concatenated using MUSCLE implemented in Geneious (Kearse *et al.* 2012). Amino acid model testing and maximum-likelihood analyses were done using RAxML v 8.2.11 (Stamatakis 2014) with 10,000 bootstrap replicates. The amino acid models JTT and WAG were used for the N- and C-terminal alignments, respectively and WAG for the concatenated alignment. FigTree v1.4.3 (http://tree.bio.ed.ac.uk/software/figtree/) was used to visualize resulting trees.

### 3D modeling of Sp_5803 terminal regions

Prediction of the tertiary structure of the terminal domains of Sp_5803 was done using the iterative threading assembly refinement (I-TASSER) server (Zhang 2008; Roy *et al.* 2010; Yang *et al.* 2015). Query sequences were threaded through resolved protein structures stored in the Protein Data Bank (PDB) and full length atomic structural models were obtained. The root mean-squared deviation score (RMSD) was used to indicate how precise a protein fits the published resolved structure, with lower RMSD indicating a high-resolution fit.

### Data availability

Genomic sequences used in this study were obtained from the WGS database under accession MWRG0000000. Supplementary material includes all data and calculations for qPCR analysis.

Supplemental material is available at figshare DOI: https://doi.org/10.25387/g3.13330910.

## Results

### *Refined annotation of silk genes from* Trichonephila clavipes

Historically, spidroins have been identified by the presence of the slowly evolving terminal domains and characteristic amino acid sequence motifs found within the repetitive region. To more easily compare the *T. clavipes* silk genes described by Babb *et al.* (2017) to previously described silk genes, we renamed them according to gene tree analyses of the terminal domains and repeat composition (Hayashi *et al.* 1999). For example, the eight major ampullate spidroin sequences previously described as MaSp-a through MaSp-h, are now more specifically categorized as either paralogs of MaSp1 or MaSp2 (Supplementary Figures S1 and S2; File S3). Moreover, spidroins that originally eluded assignment to the known spidroin classes are re-assigned as follows: Sp-74867 and Sp-907 are named MaSp3_A and MaSp3_B, respectively. The MaSp3 designation is based on the presence of C-terminal region motifs as well as poly-alanine (“poly-A”) and glycine-glycine-arginine (“GGA”) repeat motifs, that are shared with *Argiope argentata* MaSp3 (AWK58636) and *Araneus diadematus* MaSp3 (AWK58637) (Collin *et al.* 2018). The distinctiveness of *T. clavipes* MaSp3_A as a separate locus from MaSp3_B is supported by phylogenetic analyses of the N- and C-terminal domain regions (Supplementary Figures S1 and S2; File S3), as well as their placement on different contigs of the *T. clavipes* genome (MWRG01).

The spidroin clades based on separate N- and C-terminal regions are also supported in a concatenated analysis of the N- and C-termini from the 24 spidroins for which both termini are known and definitively linked based on genomic assembly (Supplementary Figure S2). Most of the complete spidroin sequences in the phylogenetic analyses are from *T. clavipes* (yellow bars in Supplementary Figure S1; 18 of the 24 sequences in Supplementary Figure S2). While the concatenated analysis generally had higher support for spidroin clades, the separate analyses make it easy to visualize the conflicting relationships implied by the different termini of the *T. clavipes MiSp* sequences, suggestive of a recombination event between *T. clavipes* MiSp_B and MiSp_C or the presence of other *MiSp* loci not yet characterized.

One of the Babb *et al.* (2017) sequences retains the “Sp” designation as it belongs to the spidroin family but could not be assigned to any known spidroin class. This is the unusual Sp_5803, which has only one canonical terminal region. The remaining four “Sp” sequences (Sp_1339, Sp_14910_A, Sp_14910_B, and Sp_8175) lack any evidence of conserved spidroin terminal regions but have amino acid compositions and repetitive organizations that are similar to spidroins. Thus, these four are renamed as “SpL” sequences, designating them as not belonging to the spidroin family (Gatesy *et al.* 2001; Garb *et al.* 2010; Chaw *et al.* 2014; Clarke *et al.* 2017; Collin *et al.* 2018; Correa-Garhwal *et al.* 2018).

### Comparison of sex-specific silk gene expression across species

**Figure 1:**
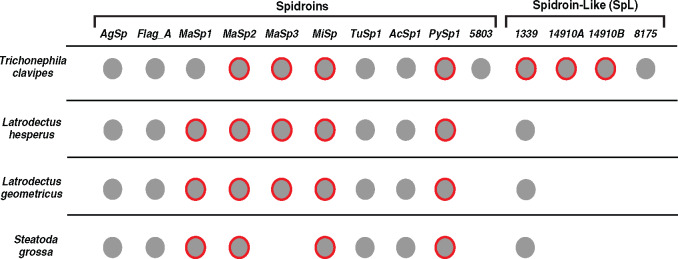
Silk gene expression of *Trichonephila clavipes*, *Latrodectus hesperus*, *Latrodectus geometricus*, and *Steatoda grossa*. Detection of expression indicated by filled circles. Silk genes significantly highly expressed in males indicated by red circles. Spidroin genes abbreviated as *AgSp1* (aggregate spidroin 1), *Flag_A* (flagelliform spidroin A), *MaSp1* (major ampullate spidroin 1), *MaSp2* (major ampullate spidroin 2), *MaSp3* (major ampullate spidroin 3), *MiSp* (minor ampullate spidroin), *TuSp1* (tubuliform spidroin 1), *PySp1* (pyriform spidroin 1), and *AcSp1* (aciniform spidroin 1). Spidroin-like sequences (*SpL*) are indicated by their annotated name. Cob-web weaver gene expression data from Correa-Garhwal *et al.* (2017).

We assessed spidroin and SpL gene expression in *T. clavipes* females and males with qPCR and compared these results to previous work on spiders from a different family, the Theridiidae (cob-web weavers) (Correa-Garhwal *et al.* 2017). We found that *T. clavipes* females express the same suite of known spidroins, except for *Sp_5803*, as females from the cob-web weaver species, *Latrodectus hesperus* (Western black widow) and *L. geometricus* (brown widow) (Figure 1). In comparisons between sexes, *T. clavipes* males express only a subset of the spidroin genes that are expressed in females, a pattern also detected in the cob-web weavers (red circles, Figure 1). For the SpL genes, we found that *SpL_1339* was expressed in all four species (*T. clavipes* and three cob-web weavers). However, the other *T. clavipes SpL* genes (*SpL_14910_A, SpL_14910_B*, and *SpL_8175*) as well as *Sp_5803* were not detected in the transcriptomes of the three cob-web weavers.

### Sex- and tissue-specific silk gene expression

**Figure 2:**
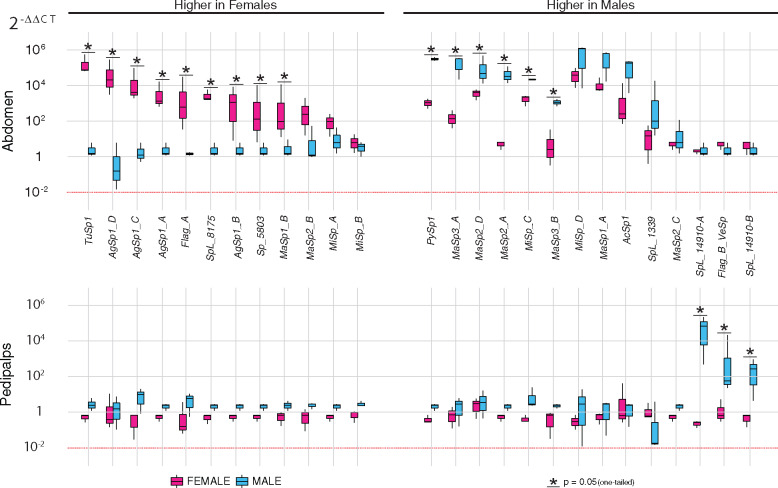
Gene expression profiles of two tissue types from male and female *Trichonephila clavipes* individuals. Box-and-whiskers plots showing the relative expression of 26 *T. clavipes* loci in individual tissue dissections (*n* = 3 biological replicates per tissue for each sex) assayed by qPCR. Tissues are depicted in horizontal panels, and include abdomen (including silk glands) and pedipalps. Loci are shown on the *x*-axis, and expression (2^−ΔΔCT^ method) is depicted on the *y*-axis (log_10_ scale). Box-and-whiskers show the range of expression values of the given gene (left of *y*-axis) relative to *RPL13a* (housekeeping gene—not shown) expression and normalized to leg tissue (*n* = 3 biological replicates per tissue for each sex). Thick black center lines represent median values. Upper whiskers represent largest observation ≤ upper quartile (Q3) +1.5 interquartile range (IQR), and lower whiskers represent smallest observation ≥ lower quartile (Q1) −1.5(IQR). Asterisks highlight loci whose expression is significantly greater (*P* = 0.05, normalized to leg tissue, one-tailed Wilcoxon rank sum tests).

For the silk gene transcripts detected in *T. clavipes* males and females (Figure 1), we quantified expression levels in different tissue types. Specifically, we evaluated the sex-specific expression in abdomen, pedipalps, and cephalothorax (Figure 2; Supplementary Figure S3). In the abdomen, which includes all silk glands, we found female spiders to have significantly higher expression of some of the spidroins associated with web-building and egg case construction (*AgSp1_A-D, Flag_A, MaSp1_B*, and *TuSp1*; asterisks in Figure 2). Female abdomens also exhibited higher expression of *Sp_5803* and *SpL_8175*. These nine silk genes that were highly expressed in females, exhibited very low expression levels in males. *T. clavipes* male abdomens were found to express significantly higher levels of *MaSp2_A, MaSp2_D, MaSp3_A-B, MiSp_C*, and *PySp1* (asterisks in Figure 2), although females did express notably high levels of these six spidroin genes.

We examined silk gene expression in tissues beyond the abdomen—where silk glands are located—across sexes. Our qPCR results confirmed that most silk genes are restricted in their expression to the abdomen, with very low expression in the pedipalps and cephalothorax, regardless of sex (Figure 2; Supplementary Figure S3). However, we observed three genes with significantly higher expression in male pedipalps, which contain the male copulatory organs that store and transfer sperm (*Flag_B_VeSp, SpL_14910_A*, and *SpL_14910_B*). One of which is the enigmatic *Flag_B_VeSp*, a spidroin closely related to *Flag_A*, but unlike *Flag_A*, is not expressed in the abdomen. Instead, *Flag_B_VeSp* is very highly expressed in the male cephalothorax and moderately expressed in the female cephalothorax (Supplementary Figure S3). Cephalothorax tissues included the venom glands, where *Flag_B_VeSp* was previously shown to be expressed in females but had not been assayed in males (Babb *et al.* 2017). The SpL sequences SpL_14910_A and SpL_14910_B lack spidroin terminal domain regions but show similarities to spidroins in having glycine rich repetitive regions. The repetitive region of SpL_14910_A has a high proportion of glycine and alanine residues arranged in repetitive motifs (Gly-Ala; “GA”) and has silk-like high molecular weight glutenin subunits (Babb *et al.* 2017). The repeat region of SpL_14910_B is rich in asparagine, glycine, and serine organized into a repeat unit that is 85 to 98 amino acids long. This repeat unit is tandemly arrayed 13 times in an arrangement similar to spidroins.

### Males have specific up- and down-regulation of silk genes

We investigated the expression profile of silk genes in more fine-scale tissue dissections of *T. clavipes* males. Again, qPCR was used to evaluate expression in these single or mixed tissue types: major ampullate silk glands, minor ampullate silk glands, other silk glands (combined aciniform and pyriform silk glands), total silk glands, combined pedipalps and legs, and venom glands (Supplementary Figure S4). We found that silk gene expression in specific male tissues to be generally consistent with the qPCR results described above (Figure 2). For example, *PySp1*, already shown to be highly expressed in abdomen (Figure 2), we observed to be expressed specifically in the combined aciniform and pyriform silk gland tissue samples (Supplementary Figure S4). Additionally, we found *MaSp1_A, MaSp2_A*, and *MaSp3_A* to be the most highly expressed silk genes in male major ampullate glands. Meanwhile, *MiSp_C* and *MiSp_D* were the highest expressed silk genes in male minor ampullate silk glands. Further, as expected, we found that *SpL_14910_A* and *SpL_14910_B* were expressed at the highest level in the combined pedipalps and legs tissue samples, and expression of *Flag_B_VeSp*, was highest in venom glands (Supplementary Figure S4).

### SpL_1339 is extremely conserved across species

**Figure 3:**
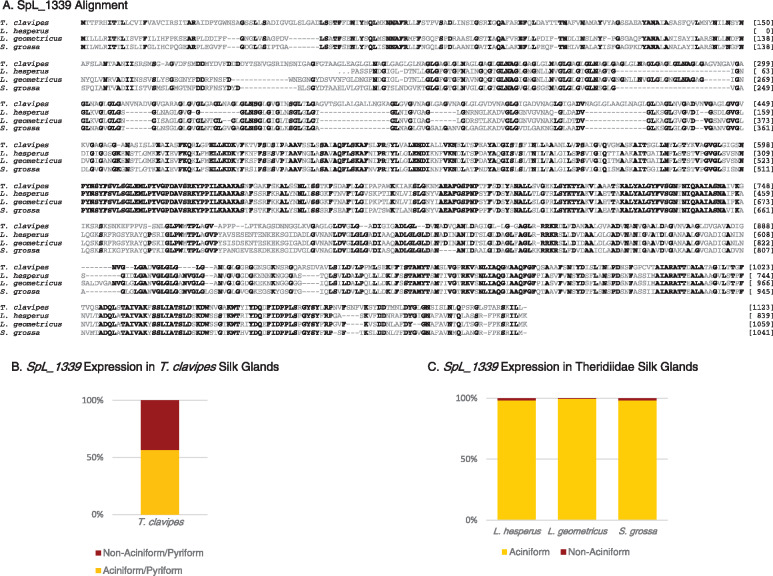
(A) Multiple sequence alignment comparing SpL_1339 of *Trichonephila clavipes*, *Latrodectus hesperus*, *Latrodectus geometricus*, and *Steatoda grossa*. Ellipsis indicate missing upstream sequence. Amino acids conserved across species shown in bold. Dashes are alignment gaps. Amino acid positions for each sequence are numbered on the right. Comparison of silk gene expression of *SpL_1339* homolog in aciniform glands and non-aciniform silk glands for (B) *T. clavipes* and (C) *L. hesperus*, *L. geometricus*, *S. grossa* in aciniform/pyriform silk glands versus non-aciniform/pyriform silk glands. Cob-web weaver gene expression data from Clarke *et al.* (2017).

While *SpL_14910_A* and *SpL_14910_B* are known from *T. clavipes* but not found in the cob-web weavers, *SpL_1339* has extensive sequence and evolutionary conservation across species (Figures 1 and 3). *SpL_1339* is not a spidroin because it entirely lacks coding sequence for the conserved spidroin N- and C-terminal regions but is an SpL because it is expressed in silk glands (Figures 1 and 2). Also, *SpL_1339* encodes a repetitive sequence consisting of silk-like motifs, including GA and GLG (Figure 3A). Sequence comparison shows that SpL_1339 has an average 50% pairwise amino acid identity with the cob-web weaver homologs and includes a remarkably uninterrupted stretch of 38 residues that is 100% conserved across species (NPYNSYFSVLSGLEMLPYVGPDAVSRKYPPILKAAKAS, Figure 3A). The perfect conservation of this 38 residue stretch is surprising because it represents maintenance for at least 170 million years (Garrison *et al.* 2016).

A striking observation from the *T. clavipes* qPCR analyses was that *SpL_1339* and two spidroin genes (*AcSp1* and *PySp1*) have similar expression patterns (Supplementary Figure S4). In both sexes, they are all highly expressed only in the combined aciniform and pyriform silk glands (males in Supplementary Figure S4; females in Babb *et al.* 2017). Moreover, the expression of *SpL_1339* was higher in the combined aciniform and pyriform silk glands than in any of the other silk glands (Figure 3B). Similarly, cob-web weaving spiders have been described as having a higher expression of *SpL_1339* in aciniform silk glands than in non-aciniform silk glands (Figure 3C). These data are compatible with the hypothesis that SpL_1339 may be specifically important for aciniform silk production, which has traditionally been associated with silks used in prey-wrapping (Hayashi *et al.* 2004; Vasanthavada *et al.* 2007).

### Sp_5803 has a unique spidroin architecture

**Figure 4:**
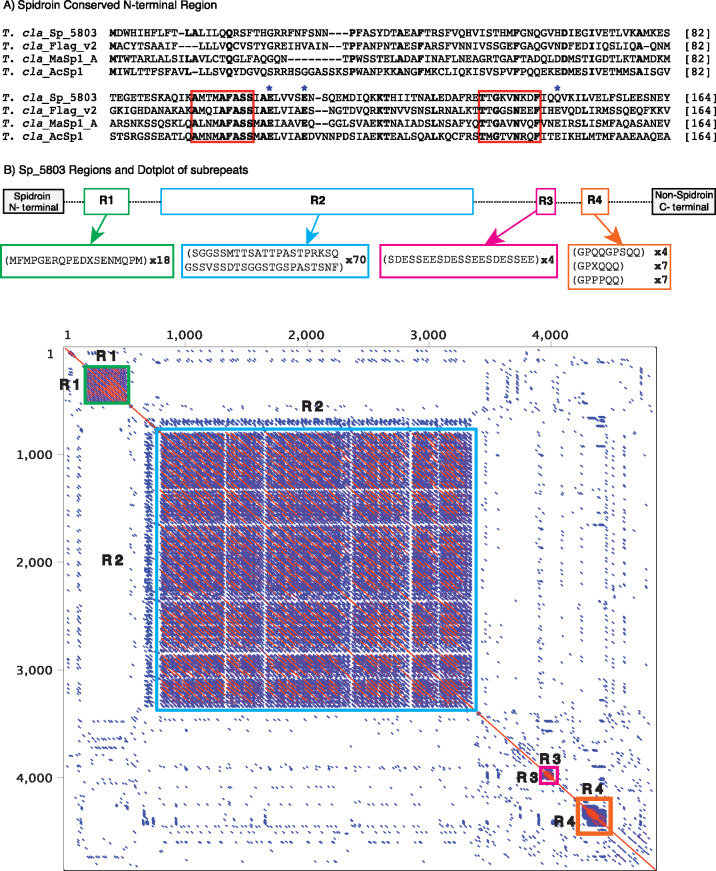
(A) Multiple sequence alignment of spidroin conserved amino (N)-terminal regions. Previously described conserved motifs are boxed in red and positions with charged residues involved in dimer lock are indicated by blue asterisks (Gao *et al.* 2013; Kronqvist *et al.* 2014; Otikovs *et al.* 2015; Atkison *et al.* 2016). (B) Regions of Sp_5803 and dot plot of the repetitive region. Dot plot shows regional self-similarity, the main red diagonal represents self-alignment. Repetitive regions within the sequence shown in boxes as follows: R1 green, R2 cyan, R3 magenta, and R4 orange. Dashes represent sequence between regions. Consensus repeat for each repeat region is shown, followed by number of repetitions within each region.

We next turned our attention to a second gene, S*p_5803*, to characterize its sequence properties in more detail. Sp_5803 has many features typical of a classic spidroin, yet is completely missing a typical spidroin C-terminal region. Sp_5803 has an N-terminal region that is the usual length of a spidroin N-terminal region (∼150 amino acid residues) (Gaines *et al.* 2010; Garb *et al.* 2010; Gao *et al.* 2013) and has the two motifs that are widely conserved across araneoid spidroin N-terminal regions, AXXXAXASS and TTGXXNXXF [“X” indicates variable amino acid position; Figure 4A, red boxes (Collin *et al.* 2018)]. Additionally, the N-terminal region of Sp_5803 has the charged glutamic acid residues that form a pH-dependent relay that is hypothesized to control the stabilization of silk dope and fiber formation (Gao *et al.* 2013; Kronqvist *et al.* 2014; Otikovs *et al.* 2015; Atkison *et al.* 2016) (Figure 4a, blue asterisks).

Following the N-terminal region, as expected for a spidroin, Sp_5803 has a large region (4846 amino acids) of repetitive sequence composed of different subrepeats (Figure 4B). The subrepeats occur in four distinct zones (depicted as colored boxes, Figure 4B: R1—green, R2—cyan, R3—magenta, and R4—orange). For example, the subrepeat SGGSSMTTSATTPASTPRKSQGSSVSSDTSGGSTGSPASTSNF is tandemly arrayed 70 times in zone R2, accounting for 57% of Sp_5803 sequence, and occurs only within that zone. Strikingly however, Sp_5803 does not have a spidroin C-terminal region. The 3′ end of Sp_5803 is complete and intact (Babb *et al.* 2017), yet, the amino acids of Sp_5803 that follow the repetitive region returned no significant BLAST hits to the nonredundant BLAST database (nr) and lack all known spidroin C-terminal conserved residues and motifs (Gao *et al.* 2013; Andersson *et al.* 2014; Collin *et al.* 2018; Strickland *et al.* 2018).

Predicted tertiary structures of both terminal regions further support that Sp_5803 has a spidroin N-terminal region but lacks a spidroin C-terminal region. The threading of the N-terminal region reconciles with published spidroin 3D structures in having five helical regions (Supplementary Figure S6A). The best match for Sp_5803 N-terminal region was *T. clavipes* Major Ampullate Spidroin 1A (PDB ID 5IZ2, RMSD 0.59). By contrast, while the predicted model of Sp_5802 C-terminal region (the last 105 aa) identified four small helical regions (Supplementary Figure S6B), the best match was not to a spidroin but to Kupe virus RNA binding protein (PDB ID 4XZC, RMSD 3.16).

## Discussion

It is generally thought that male spiders do not rely on silk as much as females. Evidence for this is that mature males have fewer silk spigots and express fewer silk genes relative to mature females (Figure 1) (Correa-Garhwal *et al.* 2017, 2018). In fact, in males, most silk genes are expressed at a lower level than in females; we show in this work that as expected, *AgSp1*, *Flag*, and *TuSp1* are expressed at a significantly lower level in males (Figure 2). Down regulation of these spidroins is consistent with reduced silk use by males, because males do not have aggregate, flagelliform, or tubuliform spigots (Moore 1977). Thus, we did not expect to find that *T. clavipes* males express six spidroins at significantly higher levels than females (Figure 2).

Two of the spidroins upregulated in males and not females are *MaSp3* genes that we found are mainly expressed in male major ampullate silk glands (Figure 2; Supplementary Figure S4). This expression pattern suggests that MaSp3 is the main protein produced in male major ampullate silk glands. By contrast, MaSp1 and MaSp2 are the main proteins in female major ampullate silk glands (Hinman and Lewis 1992; Beckwitt and Arcidiacono 1994; Ayoub *et al.* 2007). The remarkable mechanical properties of major ampullate silk fibers are attributed to the combination of MaSp1 and MaSp2 proteins, their arrangement, and their abundance. For example, the combination of amino acid motifs in MaSp1 and MaSp2 proteins creates an arrangement of crystallites and ß-sheets that influence the mechanical behavior of fibers (Xu and Lewis 1990; Hayashi *et al.* 1999; Swanson *et al.* 2006). The combination of amino acid motifs in MaSp3 is different from those found in MaSp1 and MaSp2. MaSp3 has a high concentration of polar amino acids mostly driven by serine and arginine, unlike the alanine and glycine rich repetitive regions of MaSp1 and MaSp2 (Ayoub *et al.* 2007; Collin *et al.* 2018). By refining the annotation of *T. clavipes* spidroins, we show that there is MaSp3 and expand the phylogenetic distribution of MaSp3 beyond the subclade within Araneidae described by (Kono *et al.* 2019). The realization that there is a third spidroin type, MaSp3, in major ampullate fibers raises questions about the role of MaSp3 and the mechanical properties in major ampullate fibers spun by males, given their high levels of *MaSp3* expression. In *Araneus ventricosus* females, it was suggested that although MaSp3 is highly abundant in dragline silk, there is no direct contribution to dragline mechanical properties (Kono *et al.* 2019). Male specific genetic, synthetic, and biophysical studies are needed to elucidate the role of MaSp3 in the mechanical properties in major ampullate fibers.

Other genes that are upregulated in males include *SpL*_*14910_A* and *SpL*_*14910_B*. These genes also show sex- and tissue-specific expression. Most spidroins and SpL sequences are known to be associated with silk gland tissue, but *SpL_14910_A* and *SpL*_*14910_B* are not expressed in silk glands. Instead, they are expressed in pedipalps, with significantly higher expression in male pedipalps than female pedipalps (Figure 2). Pedipalps are the intromittent organs of male spiders, functioning as sperm storage and delivery systems (Michalik and Rittschof 2011). This tissue-specific expression suggests that *SpL_14910_A* and *SpL_14910_B* could be expressed in spider sperm cells, perhaps playing a role as structural proteins in the sperm flagella.

As with SpL_14910_A and SpL_14910_B, the functional significance of any SpL sequence is poorly known. The SpL with the most compelling evidence for having a role in silk production is SpL_1339. SpL_1339 is similar to a spidroin in amino acid composition, repetitive region structure, and expression pattern, but entirely lacks spidroin terminal regions (Figure 3). SpL_1339 is remarkably conserved in sequence across species (Figure 3A), exhibiting greater conservation than even AcSp1 or PySp1, two spidroin types noted for the relative ease of aligning their respective repetitive regions across species (Ayoub *et al.* 2013; Chaw *et al.* 2014, 2016). For a region of 300 amino acids that are easily aligned across four spider species, SpL_1339 has an average pairwise amino acid identity of 77%, which is over three times as conserved as PySp1 repeats from the same four species (23%). Similarly, SpL_1339 is nearly twice as conserved as AcSp1 repeats (77% vs. 40% average pairwise identity over 300 amino acids from the same four species). The evolutionary conservation of SpL_1339 is even more striking when extending the comparison to homologs from the more distantly spiders, *Dolomedes triton* (52% similarity) and *Tengella perfuga* (47%) (Supplementary Figure S5A). These substantial levels of sequence similarity are noteworthy given that these species are estimated to have diverged from *T. clavipes* over 200 million years ago (Garrison *et al.* 2016).

The expression pattern of *SpL_1339* is also conserved across species. Comparison of *SpL_1339* expression in different silk glands shows highest expression of *SpL_1339* in aciniform/pyriform silk glands in *T. clavipes* (Figure 3B) and in aciniform silk glands in cob-web weavers (Figure 3C). Thus, *SpL_1339* expression appears specific to aciniform silk glands. Yet, how widespread *SpL_1339* is across spider diversity and how conserved it is in sequence, expression, and function remain unknown. It is clear, however, that *SpL_1339* is indeed a silk gene, and is more conserved in sequence across species than any spidroin, suggesting that *SpL_1339* is under strong selection for an essential function in the production of aciniform silk.

In contrast to *SpL_1339*, *Sp_5803* is not shared across *T. clavipes* and cob-web weavers (Figure 1). *Sp_5803*, the unusual golden orb-weaver spidroin that lacks a conserved spidroin C-terminal region, was not only found in the genome but was shown to be highly expressed by females in flagelliform glands while males had a negligible expression in all assayed tissues (Figure 2;Supplementary Figure S3). Based on its flagelliform silk gland expression pattern, *Sp_5803* appears to be associated with capture webs, and thus foraging.

Sp_5803 transforms the conventional view of spider silk proteins. The dogma in the spider silk literature is that spidroins possess conserved terminal regions that flank both sides of a central region of repetitive motifs. The observation of a conserved spidroin terminal region was noted in 1992, with the discovery of the second known spidroin family member, MaSp2 (Hinman and Lewis 1992; Beckwitt and Arcidiacono 1994). Since then, it has been routine to identify and annotate spidroins based on their terminal region sequences, as we have done (Supplementary File S3; Figures S1 and S2). However, *T. clavipes* Sp_5803 has a spidroin N-terminal region, a repetitive region comparable to spidroins, and is expressed in silk glands, but lacks any trace of a spidroin C-terminal domain (Figure 4). Given this combination of features, Sp_5803 is either a spidroin that lost its conserved spidroin C-terminal domain or is a non-spidroin that independently acquired spidroin elements. We hypothesize that the former is a simpler explanation and thus posit that Sp_5803 is indeed a spidroin, changing the dogma that spidroins are identified, in part, by their conserved C-terminal domains.

The N-terminal region includes conserved amino acid motifs that fold into the alpha helices that are posited to be involved in storage and assembly of spidroins (Askarieh *et al.* 2010; Gaines *et al.* 2010; Atkison *et al.* 2016). Thus, we propose that the Sp_5803 N-terminal region, which closely fits *T. clavipes* MaSp 1A (PDB ID 5IZ2, RMSD 0.59), is also likely to function for the storage and assembly of spidroins. Yet, because it lacks a spidroin C-terminal domain, Sp_5803 likely does not have the same mechanism of C-terminal domain promoted fiber formation, as has been described for spidroins such as MaSp and MiSp (Hedhammar *et al.* 2008; Gao *et al.* 2013; Collin *et al.* 2018; Strickland *et al.* 2018). In fact, different roles have been implicated for the C-terminal domains from different spidroin types (Ittah *et al.* 2007; Lin *et al.* 2009; Heim *et al.* 2010; Gao *et al.* 2013; Wang *et al.* 2014), and recombinant spidroin constructs have been shown to assemble into fibers without a C-terminal domain [*e.g.*, recombinant AcSp and TuSp (Lin *et al.* 2009; Wang *et al.* 2014)]. Hence, Sp_5803 appears to be an extreme demonstration of the greater conservation of the spidroin N-terminal region compared to the C-terminal region across the spidroin family (Garb *et al.* 2010).

## Conclusions

By integrating evidence from the golden orb-weaver genome, sex-specific and tissue-specific qPCR, phylogenetic analyses, and comparisons with silk genes and expression patterns in additional spider species, we enrich the functional understanding of spider silk genes (Figures 1–4). Spidroins are the most-studied spider silk proteins and have been defined by their conserved terminal regions and large repetitive regions, which tend to vary greatly between paralogs. We show that in addition to spidroins that fully conform to the classical spidroin architecture, *T. clavipes* has one deviating spidroin (*Sp_5803*) and at least four SpL sequences (*SpL_1339, SpL_8175, SpL_14910_A*, and *SpL_14910_B*). Sp_5803 has a spidroin N-terminal domain, spidroin repetitive region, and silk gland specific expression, but entirely lacks the otherwise conserved spidroin C-terminal domain. This finding indicates that the C-terminal domain is not essential in fiber formation, challenging the understanding of how spidroins have traditionally been identified and the role that the C-terminal domain plays in silk assembly (reviewed in Collin *et al.* 2018). The SpL sequence, *SpL_1339* is expressed, like some spidroins, in the small silk glands of male and female spiders. SpL_1339 shows remarkable sequence conservation across species, greater than that observed for spidroins, suggesting an essential role in spider silk production. This means that the most evolutionarily and functionally conserved structural protein in spider silk may not be a spidroin.

Although silk gene expression is generally thought to be restricted to silk glands, one spidroin violates this paradigm. *Flag_B_VeSp* is expressed in non-silk gland tissues, namely venom glands and male pedipalps. Two SpL genes, *SpL_14910_A* and *SpL_14910_B*, also have highest expression in male pedipalps and thus may be reproductive proteins. Intriguingly, *Flag_B_VeSp*, *SpL_14910_A*, and *SpL_14910_B* are species-specific, currently only known from *T. clavipes.* These genes are either of relatively recent origin or have evolved so rapidly to have obscured their homology in other species. Our findings provide clues into the roles that spidroin terminal domain regions play in the evolution and functionality of silk genes and the implications for spider silk extraordinary biomechanical properties.
